# Genome Dynamics of Campylobacter jejuni in Response to Bacteriophage Predation

**DOI:** 10.1371/journal.ppat.0030119

**Published:** 2007-08-24

**Authors:** Andrew E Scott, Andrew R Timms, Phillippa L Connerton, Catherine Loc Carrillo, Khairul Adzfa Radzum, Ian F Connerton

**Affiliations:** Division of Food Sciences, University of Nottingham, Loughborough, United Kingdom; The Pennsylvania State University, United States of America

## Abstract

Campylobacter jejuni is a leading cause of food-borne illness. Although a natural reservoir of the pathogen is domestic poultry, the degree of genomic diversity exhibited by the species limits the application of epidemiological methods to trace specific infection sources. Bacteriophage predation is a common burden placed upon C. jejuni populations in the avian gut, and we show that amongst C. jejuni that survive bacteriophage predation in broiler chickens are bacteriophage-resistant types that display clear evidence of genomic rearrangements. These rearrangements were identified as intra-genomic inversions between Mu-like prophage DNA sequences to invert genomic segments up to 590 kb in size, the equivalent of one-third of the genome. The resulting strains exhibit three clear phenotypes: resistance to infection by virulent bacteriophage, inefficient colonisation of the broiler chicken intestine, and the production of infectious bacteriophage CampMu. These genotypes were recovered from chickens in the presence of virulent bacteriophage but not in vitro. Reintroduction of these strains into chickens in the absence of bacteriophage results in further genomic rearrangements at the same locations, leading to reversion to bacteriophage sensitivity and colonisation proficiency. These findings indicate a previously unsuspected method by which C. jejuni can generate genomic diversity associated with selective phenotypes. Genomic instability of C. jejuni in the avian gut has been adopted as a mechanism to temporarily survive bacteriophage predation and subsequent competition for resources, and would suggest that C. jejuni exists in vivo as families of related meta-genomes generated to survive local environmental pressures.

## Author Summary

Campylobacter jejuni is the major cause of bacterial food-borne illness worldwide. Predation of C. jejuni by virulent bacteriophage offers the prospect of controlling bacterial populations at source in poultry. We report that in chickens, bacteriophage resistance is infrequent because the mutants that escape bacteriophage are not proficient in poultry colonisation but readily revert back to colonisation-proficient phage-sensitive types. Bacteriophage resistance is generated by reversible genomic scale inversions, leading to the activation of an unrelated bacteriophage integrated into the bacterial genome. These data not only suggest that bacteriophage therapy of C. jejuni would remain a sustainable measure to reduce poultry contamination but also demonstrate how bacterial genomes can evolve under the strong and widespread pressure of bacteriophage predation in the environment.

## Introduction

The Gram-negative bacterium Campylobacter jejuni is now recognised as a major cause of human gastroenteritis worldwide [1] and has been linked to serious neurological sequelae such as Guillain–Barré syndrome and Miller–Fisher syndrome [2]. Poultry are considered a major source of C. jejuni infections in humans, though numerous other risk factors have been proposed, including the consumption of pork, barbequing, living or working on farms, working in slaughterhouses, seasonal changes in flying insect populations, travel abroad, and the consumption of raw milk [3–8].

Targeted control of food-borne pathogens generally requires identification of the major route of transmission and thereby the most effective place to control infection. For *C. jejuni,* however, the ubiquitous presence of the organism in the environment, and the sporadic nature of the disease, coupled with the inherent genetic heterogeneity, make the task of tracing of individual strains, and thereby the source of infection, extremely difficult [9,10].

The molecular mechanisms behind this extensive diversity are not fully understood. However, C. jejuni exhibits slip-strand mutation within homopolymeric tracts, which is thought to alter the expression of a significant number of genes [11]. The majority of these genes have been identified as being involved in the production of surface structures, including key fitness determinants such as motility [12–14] and lipo-oligosaccharide synthesis [15]. C. jejuni is also known to be naturally competent under environmental conditions [16,17], though analyses of multi-locus sequence typing (MLST) profiles indicate that short lengths of DNA (less than 3 kb) are involved [18,19]. Intra-genomic recombination has been observed in C. jejuni [20,21] and C. fetus [22–24] but these events are reported to be highly localised and limited in size. Larger scale intra-genomic recombination events leading to genome diversity have, however, been reported for a wide range of other bacterial species [25–31].

As part of a study to investigate bacteriophage therapy and its impact on *Campylobacter* populations in poultry, we report that chromosomal inversions of up to 590 kb that include the origin of replication of C. jejuni arise in response to exposure to virulent bacteriophage. These inversions are associated with bacteriophage resistance, an inability to colonise chickens without reversion to bacteriophage sensitivity, and the production of a functional Mu-like bacteriophage. These data have profound implications with respect to the evolution of pathogen genomes under the strong and widespread pressure of bacteriophage predation in the environment, and the propagation of prophage under conditions in which host populations are falling.

## Results

### Isolation of Variants of C. jejuni HPC5

**Figure 1:**
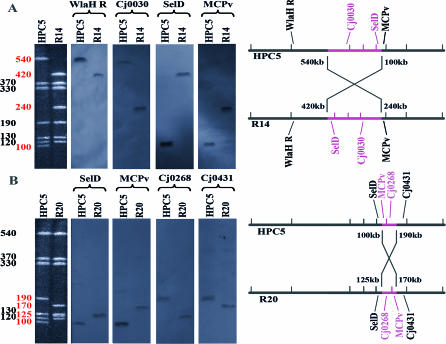
Rearrangements of C. jejuni upon Exposure to Bacteriophage PFGE and Southern hybridisations of SmaI-digested C. jejuni genomic DNA from (A) HPC5 and bacteriophage-resistant derivative R14 and (B) HPC5 and bacteriophage-resistant derivative R20. The left-hand panels show SmaI fragments of genomic DNAs resolved by PFGE, and the adjacent panels show the corresponding Southern blots hybridised to the genes indicated above each panel. The sizes of the DNA bands resolved by PFGE in kbp are indicated to the left of the image with the invariant bands marked in black and those involved in the genome rearrangements marked in red. Hybridisations were performed using DIG-labelled probes synthesised by PCR. To the right of these are scale diagrams indicating the genome rearrangements from HPC5 to generate R14 and R20. SmaI sites are indicated as vertical lines. The locations of the genes used in the hybridisations are indicated. The genes that retain their relevant positions are marked in black, and those involved in the genome rearrangements are marked in red. Images of the hybridisations using genes adjacent to the other SmaI sites can be found in Figure S1.

Following bacteriophage CP34 treatment of chickens colonised by C. jejuni HPC5, a series of CP34-insensitive isolates were recovered and examined by Loc Carrillo et al. [32]. The frequency of resistance was found to be 4% from the intestinal contents of these birds. Pulsed-field gel electrophoresis (PFGE) analysis of these isolates following SmaI digestion indicated that a number of these strains had novel PFGE macro-restriction profiles (MRPs) compared to the parent strain HPC5, though some bands were clearly related. Two novel PFGE-MRPs were observed to be common, and an example of each type was selected for further analysis. These two isolates, R14 and R20, shared five of seven SmaI bands with HPC5, but contained two novel bands of approximately 420 kb and 240 kb in R14 and 170 kb and 125 kb in R20 (Figures 1 and S1). The combined size of the novel bands in R14 and R20 was approximately equal to those of the missing bands from HPC5, indicating that gross loss or gain of genetic material was unlikely.

To rule out the possibility of contamination, strains R14 and R20 were analysed by MLST. They were found to have identical MLST profiles to HPC5 (type 356), thus confirming their origin. R14 and R20 had indistinguishable growth characteristics in vitro compared to HPC5, where the resistant phenotypes were stable for five passages representing at least 100 generations. To examine the stability of R14 and R20 in vivo, the strains were administered to chickens in the absence of bacteriophage CP34. The colonisation potentials of R14 and R20 after 5 d following administration of log_10_ 8 colony-forming units (CFU) of each strain were determined to be log_10_ 7.1 (±0.5) CFU g^−1^ for R14 and log_10_ 6.7 (±0.3) CFU g^−1^ for R20, not significantly different from that of HPC5 (log_10_ 6.9 [±0.2] CFU g^−1^). However, of the recovered isolates tested, almost all (98%) had reverted to bacteriophage sensitivity (*n* = 100). Clearly, the resistance phenotype has a large fitness cost associated with it such that it is rapidly out-competed in chickens by a relatively low initial number of sensitive revertants.

PFGE of revertant strain DNAs demonstrated all to possess MRPs different from those of their respective parent strains, where the SmaI fragments involved were those that discriminated R14 or R20 from HPC5 to link the phenotype of bacteriophage sensitivity to the observed genomic changes (Figure S2). The MRPs of the revertant strains fell into several distinct classes, termed R14-A, R14-B, R20-A, R20-B, and R20-C, with reference to their parent strains. There was an unequal distribution of the MRPs, with a preponderance of R14-B (75% of R14 revertants) and R20-A isolates (80% of R20 revertants).

To determine whether similar genomic alterations could be observed in vitro, a number of growth curves were performed using C. jejuni HPC5 and bacteriophage CP34. After 24 h of growth, 91% of the isolates were resistant to CP34 (*n* = 148). However, genomic alterations were not observed in any of these, and it is assumed that resistance arose through a different mechanism, such as point mutation of the receptor. Motility assays indicated that these strains were essentially non-motile, identifying the flagella or motility as being involved in resistance. In contrast, strains R14 and R20 derived in vivo were as motile as the parent strain HPC5.

**Figure 2:**
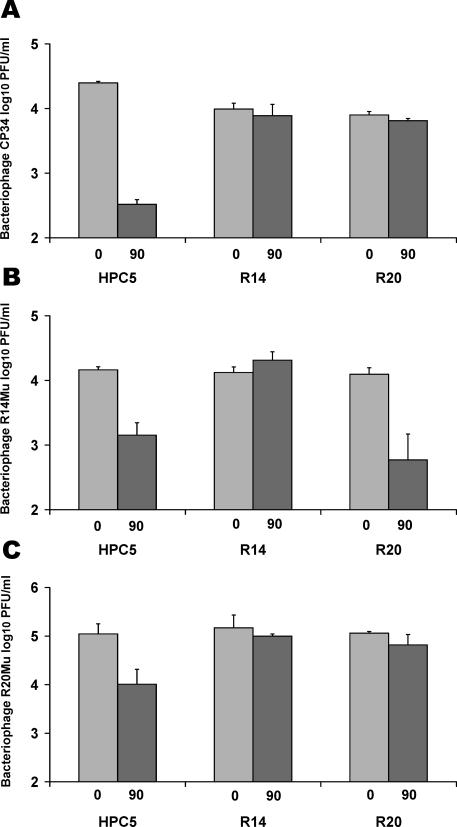
Binding Assay of Bacteriophage CP34, R14-CampMu, and R20-CampMu to C. jejuni HPC5, R14, and R20 Graphs indicating the reductions in bacteriophage titre following a 90-min incubation with C. jejuni HPC5, R14, and R20 at 42 °C with 100 rpm shaking. Error bars represent ±1 standard deviation. The top graph shows the interaction with virulent bacteriophage CP34, the middle graph shows the interaction with temperate phage R14-CampMu, and the bottom graph shows the interaction with temperate phage R20-CampMu.

Binding assays were performed to determine whether the resistance observed in R14 and R20 was receptor mediated or an abortive infection. Bacteriophage CP34 was capable of binding to HPC5, exhibiting a 98.7% drop in titre following a 90-min period of co-incubation. When incubated with either R14 or R20, however, CP34 showed no reduction in titre at the end of the 90-min period (Figure 2A).

### Reduced Colonisation Fitness Associated with Resistance to Bacteriophage

**Figure 3:**
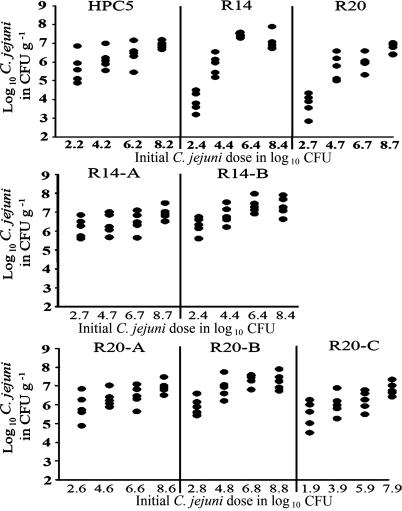
Colonisation of Broiler Chickens by Variant C. jejuni Dose response of chickens to colonisation by C. jejuni HPC5 and its daughter strains. Each data point represents the mean cecal colonisation value (log_10_ CFU g^−1^ of cecal contents) of three determinations from a single broiler chicken 48 h after administration of the campylobacters. The doses given to each bird are indicated along the *x*-axis, with five birds used per group.

Initial colonisation experiments indicated that the bacteriophage-resistant strains R14 and R20 were compromised in their ability to colonise broiler chickens because they were found to revert to bacteriophage sensitivity. This was characterised further by examining the colonisation response of broiler chickens to a range of *Campylobacter* doses for each strain (Figure 3). These data show that when administered at higher doses, all of the strains tested achieved similar colonisation values at 48 h. However, at the lowest doses of log_10_ 1.9 CFU and log_10_ 2.8 CFU, the mean cecal colonisation values of R14 and R20 were determined to be log_10_ 3.9 (±0.5) CFU g^−1^ and log_10_ 3.7 (±0.6) CFU g^−1^, respectively. These were significantly different (*p* = 0.003 and *p* = 0.002, respectively) from those of the parent strain HPC5 (log_10_ 5.7 [±0.7] CFU g^−1^) and the revertant strains R14-A (log_10_ 6.3 [±0.5] CFU g^−1^), R14-B (log_10_ 6.3 [±0.3] CFU g^−1^), R20-A (log_10_ 5.8 [±0.7] CFU g^−1^), R20-B (log_10_ 5.7 [±0.5] CFU g^−1^), and R20-C (log_10_ 5.4 [±0.6] CFU g^−1^).

### Intra-Genomic Recombination as the Source of the MRP Changes

To identify the elements responsible for the MRP changes observed in the genomes of the HPC5 derivatives, a series of restriction maps were created for these strains. SmaI sites were located by the PCR amplification of genomic DNAs using primers designed on the basis of the SmaI sites present in the genome of C. jejuni NCTC11168, followed by digestion of the PCR product with SmaI. The presence of SmaI sites within specific gene sequences were found not to vary between strains, and thus point mutation within the SmaI sites was discounted as the reason for the MRP changes. Once identified, digoxigenin (DIG)-labelled probes for the genes immediately adjacent to the SmaI sites were created along with probes spanning the SmaI sites and used for Southern hybridisation against transferred SmaI-PFGE DNA. This essentially created a range of SmaI restriction maps. Comparison of these maps for the various strains indicated that the genomes were essentially co-linear, but could be divided into sections. The polarity of these sections with respect to each other varied between the strains, indicating that genomic rearrangements involving considerable regions of the genome had occurred (up to 590 kb in R14 and 220 kb in R20). The polarities of the 540-kb and 100-kb SmaI fragments present in the R14 change and the 190-kb and 100-kb SmaI fragments in the R20 change were reversed (Figure 1). Since the SmaI sites are asymmetrically distributed, this affected the observed MRP. It is noticeable that the generation of R14 involves a rearrangement about the origin of replication (located within the 130-kb SmaI fragment), whilst the rearrangement to generate R20 does not.

**Figure 4:**
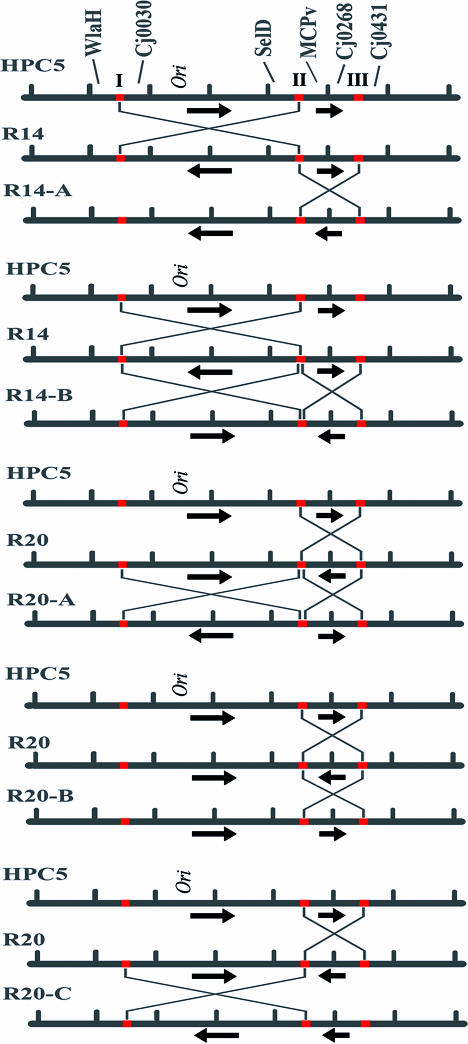
Rearrangements Involved in Altering Sensitivity to Bacteriophage Diagrammatic representation of the rearrangements involved in generating the genome configurations of the R14 and R20 revertant strains. The lineage of each of the five revertant strains is traced from C. jejuni HPC5. The locations of the CampMu prophage used as the recombination substrates are indicated in red and the direction of the variable genome segments are indicated by arrows relative to the parent strain HPC5. The identity of each prophage is indicated by I, II, and III. SmaI sites are indicated as vertical lines. This diagram is not to scale. Southern hybridisations of the PFGE of SmaI-digested C. jejuni genomic DNAs of R14, R20, and their derivatives can be found in Figure S2.

This procedure was similarly carried out on the R14-A, R14-B, R20-A, R20-B, and R20-C strains to demonstrate that these strains had undergone further genomic rearrangements, all utilising the SmaI-PFGE bands that were observed to change in the R14 and R20 genomic profiles (Figures 4 and S2). However, the rearrangements observed were not all simple reversions to the HPC5 MRP; rather, the most common isolates (R14-B and R20-A) were the result of two separate events. These data indicate that the HPC5 lineage contains three genomic locations capable of recombining with each other. Free recombination between these locations would result in eight genome configurations derived from HPC5. The strains described here represent four of eight of these potential arrangements. All of the rearrangements involve a central location within the 100-kb SmaI fragment of HPC5, which limits the permutations possible to four, all of which are observed. This central location appears key to the generation of the counter-selective phenotypes of bacteriophage resistance and inefficient chicken colonisation that are selected upon exposure to virulent bacteriophage.

### Identifying the Sites of Recombination

**Figure 5:**
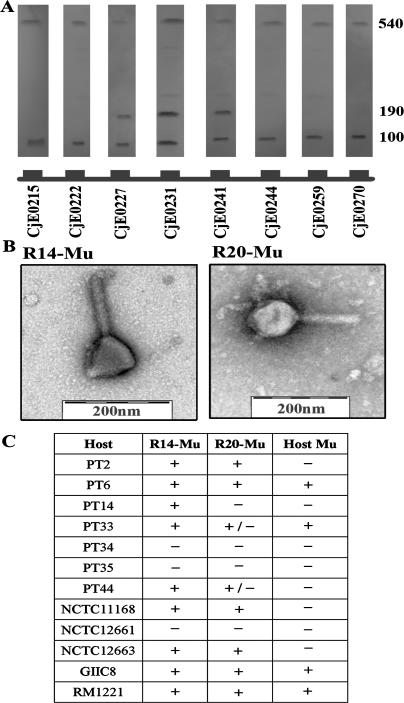
Presence and Recovery of Infectious CampMu Bacteriophage (A) Linear representation of the CampMu prophage from RM1221 with hybridisations against SmaI-digested PFGE-separated HPC5 genomic DNA using PCR-synthesised DIG-labelled probes for the genes indicated. The presence of a partial copy of the CampMu prophage in the 190-kb SmaI fragment containing genes CjE0225 to CjE0243 can be observed. (B) Transmission electron micrographs of the CampMu bacteriophage particles recovered from R14 (left) and R20 (right). The bacteriophages have icosahedral heads of 96–101 nm in diameter and tails 127–131 nm long by 21–25 nm wide. (C) The host range of R14-CampMu and R20-CampMu as determined by replication in liquid culture. The presence of CampMu prophage genes within the host (indicated by the “Host Mu” column) was determined by PCR on *Campylobacter* genomic DNAs using primers for genes CjE0222, CjE0233–CjE0237, and CjE0259 (Figure S9); and hybridisation of DIG-labelled probes of CjE0213, CjE0241, and CjE0259 to SmaI-digested PFGE-separated genomic DNAs (Figure S10).

To identify the sites of recombination, a system of chromosome walking using long-range PCR, Southern hybridisation, and direct sequencing from genomic DNA was developed. Using the genes adjacent to the SmaI sites as an anchor, long-range PCR was performed with the C. jejuni NCTC11168 genome as a guide. Once linked by PCR, DIG-labelled probes were created for individual genes to determine in which SmaI-PFGE band the gene was located. In places where the gene order in the HPC5 lineage diverged from that of NCTC11168, a system of sequencing directly from genomic DNA and inverse PCR was employed to determine the identity of the adjacent sequences. These DNA sequences appear in Figures S3–S8. The rearrangement end points were determined to be within copies of Mu-like prophages, similar to the prophage identified in C. jejuni RM1221 [33]. However, whereas RM1221 contained a single copy of the prophage, HPC5 and its daughter strains contained two complete copies and a partial copy (ORFs CjE0227 to CjE0241) at distinct genomic locations. In HPC5, the complete copies are between the 3′-end of Cj1470 (*CtsF*) and the 5′-end of Cj0167c in the 540-kb SmaI fragment (designated CampMu-I), and between the 3′-end of Cj0167 and the 3′-end of Cj1470c (*CtsF*) in the 100-kb SmaI fragment (CampMu-II), resulting in the disruption of both *CtsF* and Cj0167c. The partial copy is between an unknown gene and a paralog of CjE0225 in the 190-kb SmaI fragment (CampMu-III). The R14 rearrangement involved recombination between the two complete copies of the CampMu prophage, whilst the R20 rearrangement involved recombination between CampMu-II and CampMu-III. Similarly, the rearrangements to create the R14 and R20 derivative strains took place within these CampMu prophage DNA sequences (Figure 5A).

### CampMu Bacteriophage

The discovery that the inversion sites featured CampMu prophage sequences led to studies of whether the CampMu lysogens could be induced to liberate bacteriophage particles. However, it was determined that both R14 and R20 were capable of producing a CampMu bacteriophage without the need for induction. Bacteriophages were produced at a rate of approximately one particle per 50 cells (R14 = 49, R20 = 61). These bacteriophages were examined by transmission electron microscopy (Figure 5B) and identified as corresponding to the CampMu prophage by PCR amplification of DNA extractions from the bacteriophage using CampMu primer pairs, but not from control C. jejuni 16s rDNA primers. Infectious CampMu bacteriophage particles could not be detected in supernatants from HPC5 or from the R14 and R20 revertant strains exhibiting phage sensitivity as tested by titration of the supernatants on all of the strains used in this study and a further panel of 139 independent C. jejuni isolates from broiler chickens, chicken meat, and humans.

The strains capable of supporting the replication of the virulent bacteriophage CP34 (HPC5 and the R14/R20-derived revertants) were also capable of supporting replication of bacteriophage R14-CampMu and R20-CampMu whilst R14 and R20 were resistant to CP34 and the bacteriophage they produce. To compare whether the resistance observed with R14-CampMu and R20-CampMu was due to the failure of the phage to bind the bacterial host in a similar way to CP34 or to abortive infection, binding assays of the CampMu phage were performed under similar conditions (Figure 2B and 2C). Bacteriophage R14-CampMu and R20-CampMu were capable of binding the progenitor host strain HPC5, exhibiting approximately 90% reductions in phage titre after a 90-min incubation. R14-CampMu showed no reduction in phage titre when incubated with the R14 strain producing it, but incubation with R20 produced a 95% fall in phage titre. R20-CampMu showed no evidence of binding to either R14 or R20.

Further evidence for differences between R14-CampMu and R20-CampMu became apparent upon testing the susceptibility of a variety of C. jejuni strains, which revealed that the CampMu bacteriophage exhibited different host ranges (Figure 5C), and that these were maintained following growth of the CampMu bacteriophage on susceptible strains not of the HPC5 lineage. It was also notable that R14-CampMu and R20-CampMu could replicate on independent strains carrying CampMu prophage genes (Figures S9 and S10).

## Discussion

One of the major fears concerning bacteriophage therapy is the potential for bacteriophage-induced genome evolution. Numerous examples exist where temperate bacteriophages are associated with virulence determinants, for example, the genes encoding the toxins of cholera, diphtheria, and verotoxigenic Escherichia coli [34–36]. However, it is generally assumed that using virulent bacteriophage will avoid this problem. This report indicates that virulent bacteriophage have the potential to activate dormant prophage, leading to rapid pathogen evolution; and via host recombination the evolution of temperate bacteriophage leading to the production of chimeric phage with novel phenotypes. However, we also show that whilst pathogen evolution can be rapid, resistance to the therapeutic bacteriophage is associated with a draconian fitness cost that renders the resistant strains non-competitive in the absence of the bacteriophage.

Clearly though, the primary benefit of bacteriophage therapy in this instance is to temporarily reduce the carriage of C. jejuni rather than to eliminate it. Indeed, the ability of C. jejuni to enter what is effectively a transient survival state is evidence of the unusual measures *Campylobacter* can employ to survive environmental pressures. Recent evidence suggests that *Campylobacter* is limited in stress response mechanisms [37] and can use genome alterations such as localised frame-shift mutations and slip-strand phase variation to modify gene expression as a substitute for the maintenance of structured regulatory mechanisms [11–15]. The evidence presented here indicates that C. jejuni can use specific genome inversions to survive adverse ecological conditions. Under these conditions, any given *Campylobacter* recovered is actually but a single representative of a larger family of related meta-genomes under continual flux, the relative proportions of which are dictated by local environmental pressures. Amongst derivate genomes are those in which the origin of replication has been inverted, which could give rise to yet wider changes in gene regulation. This form of chaotic genome regulation is a striking example of the extraordinary strategies adopted by C. jejuni to survive. This type of genomic scale regulation would also suggest that complementary typing methods are required to adequately differentiate C. jejuni strains; methods should be selected that sample the whole genome in parallel with those that are highly discriminatory for smaller sections of the genome. In this example, strains R14 and R20 cannot be differentiated from HPC5 by MLST alone despite the large phenotypic differences observed. A combination of MLST and PFGE methods are required to distinguish these strains and identify them as being different but closely related.

Intra-genomic rearrangements have been reported previously for the flagellin locus of C. jejuni [20,21] and the *sap* locus of C. fetus [22–24]. However, these rearrangements are relatively short (<5 kb) and highly localised, utilising areas of sequence homology (flagellin) or specific recombination pathways (*sap* locus). The genome sequence data available for C. jejuni are notable for their lack of repeated sequences, and the completed genomes of NCTC11168 and RM1221 are essentially co-linear; therefore, it would not be unreasonable to suggest that genome rearrangements of C. jejuni are either limited or, given the idea of *Campylobacter* as a meta-genomic organism, that the observed genome organisations are optimal for in vitro cultures*.* However, changes to PFGE-MRPs have been noted elsewhere [17,38,39], indicating that chromosomal rearrangements are possible for strains carrying repeated sequences as substrates for homologous recombination such as the prophage sequences documented here.

The observation that the R14 and R20 rearrangements occur in vivo rather than the generation of resistance through mutation of the receptor or a specific binding component is likely a consequence of the essential nature of these components. The frequency at which bacteriophage-resistant mutants are generated in vitro (91%) with HPC5 suggests that there are easier paths to escape bacteriophage predation. However, all the mutants selected in vitro were impaired in motility. Flagella components have been demonstrated to be dominant colonisation factors [40–42], and thus it is not surprising that resistant isolates lacking motility do not survive long in chickens. Bacteriophage CP34 appears to have selected an essential component of the bacteria's intestinal lifecycle, where dense host populations are likely to be most abundant. C. jejuni flagellin is known to be polymorphic and variably glycosylated, leading to differences in sero-specificity [43–46]. Bacteriophage predation may be the direct driving force behind the development of such antigenically variable flagellins rather than host immune evasion, as considered previously [47].

The spontaneous production of CampMu bacteriophages following bacteriophage therapy is of concern because Mu bacteriophages are potential agents of mutation. However, the influence of potential mutator phage needs to be considered against the mutation-driven lifestyle of *C. jejuni,* which does not carry a full complement of DNA repair mechanisms in the expectation that genomic variation will modify gene expression to overcome adverse conditions. Moreover, evidence suggests that *Campylobacter* populations are already exposed to CampMu bacteriophage through the mechanism outlined here. Virulent bacteriophages of the family Myoviridae, like CP34, are common in chickens harboring campylobacters. Isolation rates of around 20% in United Kingdom conventional broiler flocks, and more frequently in environmentally exposed free-range and organic flocks, have been reported [48–50]. A recent survey of C. jejuni and C. coli isolates found that 19 of 67 and two of 12 of the respective isolates contained at least one prophage gene [51]. This corresponds well with the four of 12 positive C. jejuni strains reported here. If these figures are representative of general *Campylobacter* populations, then the likelihood is that these processes are quite common.

The recombination events leading to the strain variants reported here are centred on a 9-kb region of DNA sequence that is shared between prophages CampMu-I, -II, and -III (genes CjE0227 to CjE0241). Recombination between CampMu-I and CampMu-II gives rise to the R14 genome that produces bacteriophage R14-CampMu, and recombination between CampMu-III and CampMu-II gives rise to the R20 genome that produces R20-CampMu. These events lead to the generation of chimeric CampMu prophage in which the genes CjE0242 to CjE0273 adjacent to the recombination resolution of R14 are exchanged, and genes CjE0215 to CjE0226 adjacent to the recombination resolution of R20 are exchanged. These exchanges enable functional excision of CampMu bacteriophages with different gene contents that are themselves distinguishable by their *Campylobacter* host range (Figure 5C).

What these events have in common is that they lead to resistance to the virulent phage CP34 that is unable to bind the host bacterium. The non-binding of CP34 may arise through two potential mechanisms: 1) changes in host surface structures that are required for phage adsorption; or 2) receptor saturation, if CP34 shares a receptor recognition site with the CampMu phages, and these sites are saturated in the R14 and R20 cultures that produce them. Changes in the surface structures of host bacteria leading to bacteriophage immunity often accompany the state of lysogeny and are mediated through the acquisition of additional genes, commonly known as morons, the control of which are generally independent of the regulation of prophage within which they are sited [52]. In the case of R14 and R20, the change in surface structure expression would have to be associated with the activation of the prophage, for although a wider set of genes other than those affected by the recombination resolution site could be influenced by the gross inversions, the second site reversion events that reinstate phage sensitivity as a consequence of the chromosome rearrangements would militate against the inversions themselves being responsible for the change in phenotype. A consequence of the prophage control of surface structures is that campylobacters carrying CampMu may be biased for certain phage types through the regulated expression of their receptors, or indeed for specific environments according to the need of the organism to express these surface structures. In the latter case, there would be strong selective pressure to inactivate the prophage even at the expense of inverting significant parts of the genome to reassert the control necessary to respond to alternative environments.

Bacteriophage R14-CampMu can bind strain R20, suggesting the receptor site for the phage is still available on this strain, but despite this the R14-CampMu phage does not form plaques on R20, indicating there is likely an underlying resistance mechanism that results in abortive infections. However, neither bacteriophage R14-CampMu nor R20-CampMu are able to bind the respective C. jejuni strains that produced them, and therefore receptor saturation remains a plausible mechanism by which R14 and R20 prevent super-infection.

Considering the above, it is of interest to contemplate how multiple prophage copies have been fixed within the HPC5 lineage. It is a general tenet that the state of lysogeny renders the bacteria immune to infection by homologous bacteriophage. Therefore, it is unlikely that the naïve HPC5 precursor was lysogenised by multiple copies of the same bacteriophage, though not of course impossible. Indeed, it is more likely that a single prophage was present and replicated itself by transposition during the first stages of prophage lytic multiplication. It is known that the position of DNA replication forks influences the location of transposition [53], and the equidistant spacing of the CampMu-I and CampMu-II copies about the origin of replication may indicate that these prophage inserted here as a result of the presence of replication forks symmetrically arranged around the origin. This does lead to the question as to why the replication of the putative CampMu phage was not carried through to completion, namely, the lysis of the host cell. A potential answer to this is a recombination event. The R14 genome structure is similar to that of C. jejuni NCTC11168 and RM1221, whereas HPC5 has a section of genome of reversed polarity. It is possible the R14 configuration represents the original bacteriophage-negative progenitor that became lysogenised by CampMu. When this prophage began to replicate by transposition, the sudden presence of extensive regions of homology allowed recombination within the genome of this strain. Presumably, this recombination led to strain HPC5, where the CampMu prophage was inactivated, and the cell survived. This is supported by the fact that the CampMu cannot be recovered from HPC5, and yet frequently exits the cell in R14 and R20, suggesting that in HPC5, the Mu is inactivated. If true, this is another example of how flexibility within the C. jejuni genome has enabled it to survive the induction of a lysogenic bacteriophage that should have resulted in cell death, and to capitalise on the outcome through evasion of virulent bacteriophage.

The genes present in the partial copy of the CampMu prophage (CampMu-III) have previously been identified as being present en masse in a variety of *Campylobacter* [51] that lack the other prophage genes (CjE0215 to CjE0226 and CjE0242 to CjE0273). Analysis of the unique sequences adjacent to CampMu-III in HPC5 indicates that these have similarity to bacteriophage genes from other sources, most notably to a phage major tail tube protein from C. jejuni 260.94. It would appear that genes CjE0227 to CjE0241 represent a module of a CampMu genome (CampMu-III) comprising a central region similar to that of the RM1221 CampMu, but flanked by novel prophage genes. Recombination between prophage genomes leads to exchange of these modules and the evolution of the prophage genome. Intra-chromosomal recombinations between the prophage in HPC5 are a direct example of such events, producing chimeric bacteriophage that can exploit differing host ranges.

## Materials and Methods

### *Campylobacter* and bacteriophage storage and growth conditions.

Campylobacters were cultured on blood agar plates (blood agar base No. 2 with 5% defibrinated horse blood; Oxoid, http://www.oxoid.com/) in gas jars under microaerobic conditions (5% O_2_, 85% N_2_, 10% CO_2_) at 42 °C for 24 to 48 h. Growth curves were conducted by inoculating log_10_ 7 CFU of the C. jejuni into 100 ml of nutrient broth No. 2 (Oxoid) and incubating at 42 °C under microaerobic conditions with 100 rpm orbital rotation.

Bacteriophage CP34 was propagated on C. jejuni HPC5 and recovered using a plate lysis method and stored at 4 °C in SM buffer [32]. Bacteriophage R14-CampMu and R20-CampMu were recovered from blood agar plate cultures of either C. jejuni R14 or R20 by swabbing into SM buffer and passage through a 0.2-μm filter to remove bacteria. Testing of *Campylobacter* strain susceptibility to bacteriophage was performed as described previously [48]. The susceptibility of *Campylobacter* strains to bacteriophage R14-CampMu and R20-CampMu was tested by growth of the appropriate *Campylobacter* strain in 100 ml of nutrient broth No. 2 in the presence of log_10_ 3 plaque-forming units (PFU) ml^−1^. Samples were recovered and bacteriophage enumerated before and after growth for 24 h at 42 °C on HPC5.

### Bacteriophage binding assay.

Bacteriophage binding assays were performed to determine whether insensitivity to bacteriophage was due to surface or intracellular factors. Overnight *Campylobacter* growth from blood agar plates was swabbed into nutrient broth No. 2, centrifuged at 13,000*g* for 1 min, and the cell pellet resuspended in nutrient broth No. 2. The cells were washed in this manner twice more and, upon final resuspension, were adjusted to contain log_10_ 10 CFU ml^−1^ as estimated from OD_600_. Bacteriophage was added at concentrations of log_10_ 4–5 PFU ml^−1^ to the *Campylobacter* suspension and incubated at 42 °C with 100 rpm shaking under aerobic conditions for 90 min. Samples were taken at 0 and 90 min, filtered through a 0.2-μm filter, and stored at 4 °C until enumeration of the bacteriophage.

### Chicken colonisation.

*Campylobacter*-free Ross broiler chickens were used to determine the colonisation of different *Campylobacter* strains in the presence and absence of bacteriophage. To ensure that the experimental birds remained free of naturally occurring infection, faeces and cloacal swabs were taken each day from hatch and tested for *Campylobacter* by direct plating on CCDA agar and for *Salmonella* by enrichment in Rappaport–Vassiliadis soya peptone broth (Oxoid), then plating on xylose-lysine desoxycholoate agar (Oxoid). Birds were dosed with *Campylobacter* at 21 d of age and with bacteriophage where applicable at 25 d of age. Following sacrifice, the ceca, upper (proximal small intestine) and lower intestines of the birds were first separated by ligature and then removed by sterile dissection. The lumenal contents were collected for *Campylobacter* and bacteriophage isolation as described previously [32].

### Multi-locus sequence typing.

MLST was performed as described previously [54] with reference to the C. jejuni MLST database (http://pubmlst.org/campylobacter/) to determine the sequence alleles.

### Pulsed-field gel electrophoresis.

PFGE was carried out on SmaI-digested genomic DNA and compared to the known profiles of the test strains [55].

### Preparation of genomic DNA, PCR, and sequencing.

*Campylobacter* DNA isolation was carried out by using GenElute Bacterial Genomic DNA purification kit (Sigma-Aldrich, http://www.sigmaaldrich.com/) or Wizard Genomic DNA purification kit (Promega, http://www.promega.com/). Bacteriophage genomic DNA isolation was performed according to standard procedure [32] using proteinase K digestion followed by phenol-chloroform extraction and precipitation.

Oligonucleotide primers were designed using the NCTC11168 and RM1221 sequences (Sigma-Genosys, http://www.sigmaaldrich.com/Brands/Sigma_Genosys.html). A list of primers used in this study can be found in Table S1. PCRs were performed in 50-μl volumes using a Techne Progene thermal cycler. Reactions consisted of 2.5 U AccuTaq DNA polymerase (Sigma-Aldrich), each dNTP at 500 μM (Promega), forward and reverse primers at 400 nM each, 2% v/v dimethyl sulphoxide, and 100–500 ng of genomic DNA as template in AccuTaq DNA polymerase buffer. DIG-labelled probes for Southern hybridisation were synthesised by PCR with the replacement of the 400 μM dTTP with dTTP at 368 μM and DIG-11-dUTP at 32 μM (Roche, http://www.roche.com/).

Sequencing of PCR products was carried out by MWG Biotech AG (http://www.mwg-biotech.com/) using the ValueRead system. Direct sequencing of genomic DNA was achieved using the same system but with 20 μg of genomic DNA prepared using the Wizard Genomic DNA purification kit.

### Southern transfer and hybridisation.

DNA fragments separated in PFGE gels were transferred to Hybond N+ nylon membranes (Amersham Biosciences, http://www.gelifesciences.com/) using the capillary method. Hybridisation probes were synthesised by PCR as described above. Hybridisations were performed overnight at 42 °C using DIG Easy Hyb. Buffer (Roche). The membranes were blocked using 1% blocking reagent (Roche) before antibody binding with 150 mU ml^−1^ anti-DIG-AP in 1% blocking reagent. Colour development was performed by incubation in 100 mM tris-HCl (pH 9.5), 100 mM sodium chloride, 0.45 mg ml^−1^ nitro-blue tetrazolium chloride, and 0.175 mg ml^−1^ 5-bromo-4-chloro-3-indolyl-phosphate, 4 toluidine salt.

### Electron microscopy.

Bacteriophage particles at log_10_ 8 PFU ml^−1^ were absorbed onto a glow-discharged carbon-coated Pioloform grid and stained with uranyl acetate. These were examined using a JEOL 100CX transmission electron microscope (http://www.jeol.com/) operating at an acceleration voltage of 80 kV.

### Motility assays.

C. jejuni strains were grown on blood agar overnight. A loop of bacteria was inoculated into the centre of a motility plate (Mueller–Hinton broth with 0.4% agar) and grown micro-aerobically for 24 h. Motility was assessed as a function of the radius of the motility halo.

## Supporting Information

Figure S1 PFGE and Southern Hybridisations of SmaI-Digested *Campylobacter* Genomic DNAs Using Probes Adjacent to the SmaI Sites (A) HPC5 and R14 and (B) HPC5 and R20. Hybridisations were performed using PCR-synthesised, DIG-labelled probes for the genes indicated above the Southern hybridisation panels and show the conservation of these SmaI fragments between strains. Adjacent to these are scale diagrams indicating the genome rearrangements from HPC5 to generate R14 and R20. SmaI sites are indicated as vertical lines. The “WlaH L” probe was obtained by gel purification of a 320 bp DNA fragment following HindIII digestion of a DIG-labelled PCR product synthesised using the “WlaH F” and “WlaH R” primers. The Southern hybridisations for genes labelled with an asterisk can be found in Figure 1.
(458 KB PDF)

Figure S2 PFGE and Southern Hybridisations of SmaI-Digested *Campylobacter* Genomic DNAs Images of genomic DNA from various *Campylobacter* strains following SmaI-digestion and PFGE separation (R14-A, R14-B, R20-A, R20-B and R20-C MRPs are shown next to HPC5, R14 and R20 from which they are derived) alongside Southern transfer and hybridisation with DIG-labelled probes for the genes indicated. These genes are located next to the SmaI sites of HPC5 which are involved in the genomic rearrangements. The “WlaH R” probe was obtained by gel purification of a 320 bp DNA fragment following HindIII digestion of a DIG-labelled PCR product synthesised using the “WlaH F” and “WlaH R” primers.
(230 KB PDF)

Figure S3 DNA Sequence Adjacent to the CampMu Prophage Gene CjE0213 in the 540-kb SmaI Fragment of C. jejuni HPC5 (A) Genomic DNA sequence adjacent to CjE0213 540-kb SmaI fragment of HPC5 identified as the 3′-end of Cj1470c. Underlined sequences represent genes identified through BLASTx.
(B) Identification of the genes by BLASTx and their putative function.
(173 KB PDF)

Figure S4 DNA Sequence Adjacent to the CampMu Prophage Gene CjE0270 in the 540-kb SmaI Fragment of C. jejuni HPC5 (A) Genomic DNA sequence adjacent to CjE0270 540-kb SmaI fragment of HPC5 identified as the 5′-end of Cj0167c. Underlined sequences represent genes identified through BLASTx.
(B) Identification of genes by BLASTx and their putative function.
(463 KB PDF)

Figure S5 DNA Sequence Adjacent to the CampMu Prophage Gene CjE0213 in the 100-kb SmaI Fragment of C. jejuni HPC5 (A) Genomic DNA sequence adjacent to CjE0213 identified as the 3′-end of Cj0167c. Underlined sequences represent genes identified through BLASTx.
(B) Identification of genes by BLASTx and their putative function.
(71 KB PDF)

Figure S6 DNA Sequence Adjacent to the CampMu Prophage Gene CjE0270 in the 100-kb SmaI Fragment of C. jejuni HPC5 (A) Genomic DNA sequence adjacent to CjE0270 identified as the 5′-end of Cj1470c. Underlined sequences represent genes identified through BLASTx.
(B) Identification of genes by BLASTx and their putative function.
(190 KB PDF)

Figure S7 DNA Sequence Adjacent to the CampMu Prophage Gene CjE0241 in the 190-kb SmaI Fragment of C. jejuni HPC5 (A) Genomic DNA sequence adjacent to CjE0241 has no significant matches in the database. Underlined sequences represent genes identified through BLASTx.
(B) Identification of the genes by BLASTx and their putative function.
(238 KB PDF)

Figure S8 DNA Sequence Adjacent to the CampMu Prophage Gene CjE0227 in the 190-kb SmaI Fragment of C. jejuni HPC5 (A) Genomic DNA sequence adjacent to CjE0227 features paralogous prophage sequences followed by a gene with no significant matches in the database. Underlined sequences represent genes identified through BLASTx.
(B) Identification of the genes by BLASTx and their putative function.
(112 KB PDF)

Figure S9 PCR Amplification of CampMu Genes from *Campylobacter* Strains Images of the PCR products obtained following PCR using the indicated *Campylobacter* genomic DNA as a template with primers for CampMu prophage genes CjE0222, CjE0233–CjE0237, and CjE0259. Primers for 16S rRNA genes were used as the positive control. Primer sequences were designed on the RM1221 genome sequence.
(106 KB PDF)

Figure S10 Hybridisation of CampMu Genes to SmaI-Digested Genomic DNAs from Various *Campylobacter* Strains Images of the genomic DNA from various *Campylobacter* strains following (A) SmaI digestion, separation by PFGE, Southern transfer to nylon membranes, and hybridisation with DIG-labelled probes for the CampMu prophage genes (B) CjE0222, (C) CjE0241, and (D) CjE0259. Indicated above the hybridisation panels are those strains identified as containing that CampMu prophage genes.
(93 KB PDF)

Table S1 Oligonucleotides Used in This Study Table indicating the PCR primers used during this study and their primary functions. All primers are designed and named based on the C. jejuni NCTC11168 or RM1221 genes they are located within. Primers used for MLST analysis (not shown here) were according to Dingle et al. [54].
(694 KB PDF)

### Accession Numbers

DNA sequences associated with this manuscript appear in the following supplementary figures with the corresponding GenBank (http://www.ncbi.nlm.nih.gov/Genbank/index.html) accession numbers: Figure S3, Cj1468-CjE0213 (EF581842); Figure S4, ORF0656-Cj0167 (EF581846); Figure S5, *miaA*-CjE0213 (EF581841); Figure S6, ORF0656-Cj1470 (EF581845); Figure S7, Unk-CjE0241 (EF581844); Figure S8, Unk-CjE0227 (EF581843).

## Abbreviations

CFU: colony-forming units
DIG: digoxigenin
MLST: multi-locus sequence typing
MRP: macro-restriction profile
PFGE: pulsed-field gel electrophoresis
PFU: plaque-forming units
