# Correction: Genome Dynamics of Campylobacter jejuni in Response to Bacteriophage Predation

**DOI:** 10.1371/journal.ppat.1011332

**Published:** 2023-04-12

**Authors:** Andrew E. Scott, Andrew R. Timms, Phillippa L. Connerton, Catherine Loc Carrillo, Khairul Adzfa Radzun, Ian F. Connerton

The fifth author’s name is spelled incorrectly. The correct name is: Khairul Adzfa Radzun. The correct citation is: Scott AE, Timms AR, Connerton PL, Loc Carrillo C, Radzun KA, Connerton IF (2007) Genome Dynamics of Campylobacter jejuni in Response to Bacteriophage Predation. PLoS Pathog 3(8): e119. https://doi.org/10.1371/journal.ppat.0030119.
