# Supplementary material for: Genome Dynamics of Campylobacter jejuni in Response to Bacteriophage Predation
Source: PLoS Pathog. 2007 Aug 24;3(8):e119. doi: 10.1371/journal.ppat.0030119 (PMC1950947; doi:10.1371/journal.ppat.0030119)
Supplement: Supplementary file 1 [file ppat.0030119.sg001.pdf]

Figure S1

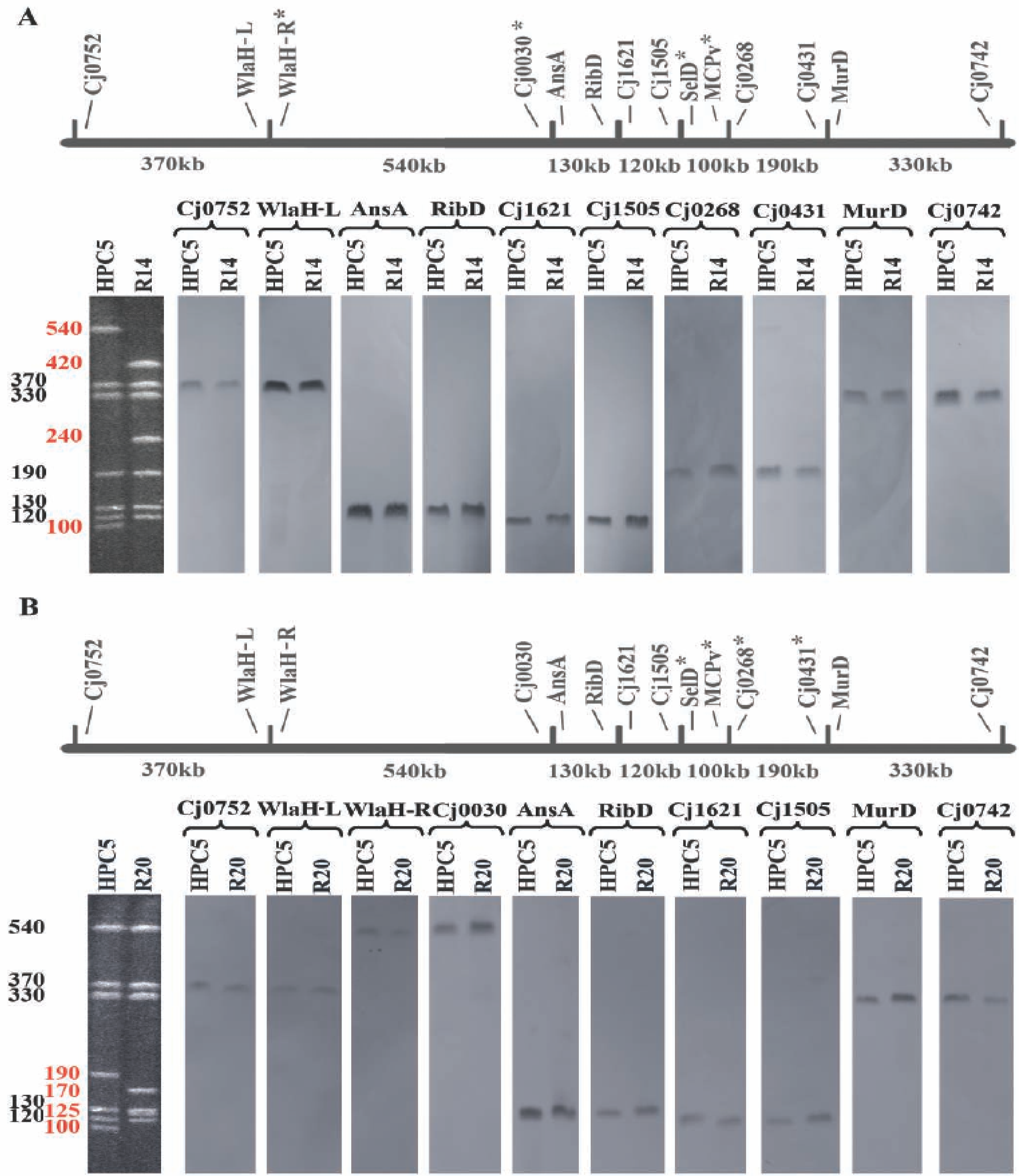
A
Cj0752
WlaH-L
WlaH-R*
Cj0030 *
AnsA
RibD
Cj1621
Cj1505
SelD*
MCPv*
Cj0268
Cj0431
MurD
Cj0742
370kb
540kb
130kb
120kb
100kb
190kb
330kb
Cj0752 WlaH-L AnsA RibD Cj1621 Cj1505 Cj0268 Cj0431 MurD Cj0742
HPC5 R14
540
420
370
330
240
190
130
120
100
B
Cj0752
WlaH-L
WlaH-R
Cj0030
AnsA
RibD
Cj1621
Cj1505
SelD*
MCPv*
Cj0268*
Cj0431*
MurD
Cj0742
370kb
540kb
130kb
120kb
100kb
190kb
330kb
Cj0752 WlaH-L WlaH-R Cj0030 AnsA RibD Cj1621 Cj1505 MurD Cj0742
HPC5 R20
540
370
330
190
170
130
125
120
100
