# Supplementary material for: Genome Dynamics of Campylobacter jejuni in Response to Bacteriophage Predation
Source: PLoS Pathog. 2007 Aug 24;3(8):e119. doi: 10.1371/journal.ppat.0030119 (PMC1950947; doi:10.1371/journal.ppat.0030119)
Supplement: Supplementary file 2 [file ppat.0030119.sg002.pdf]

Figure S2

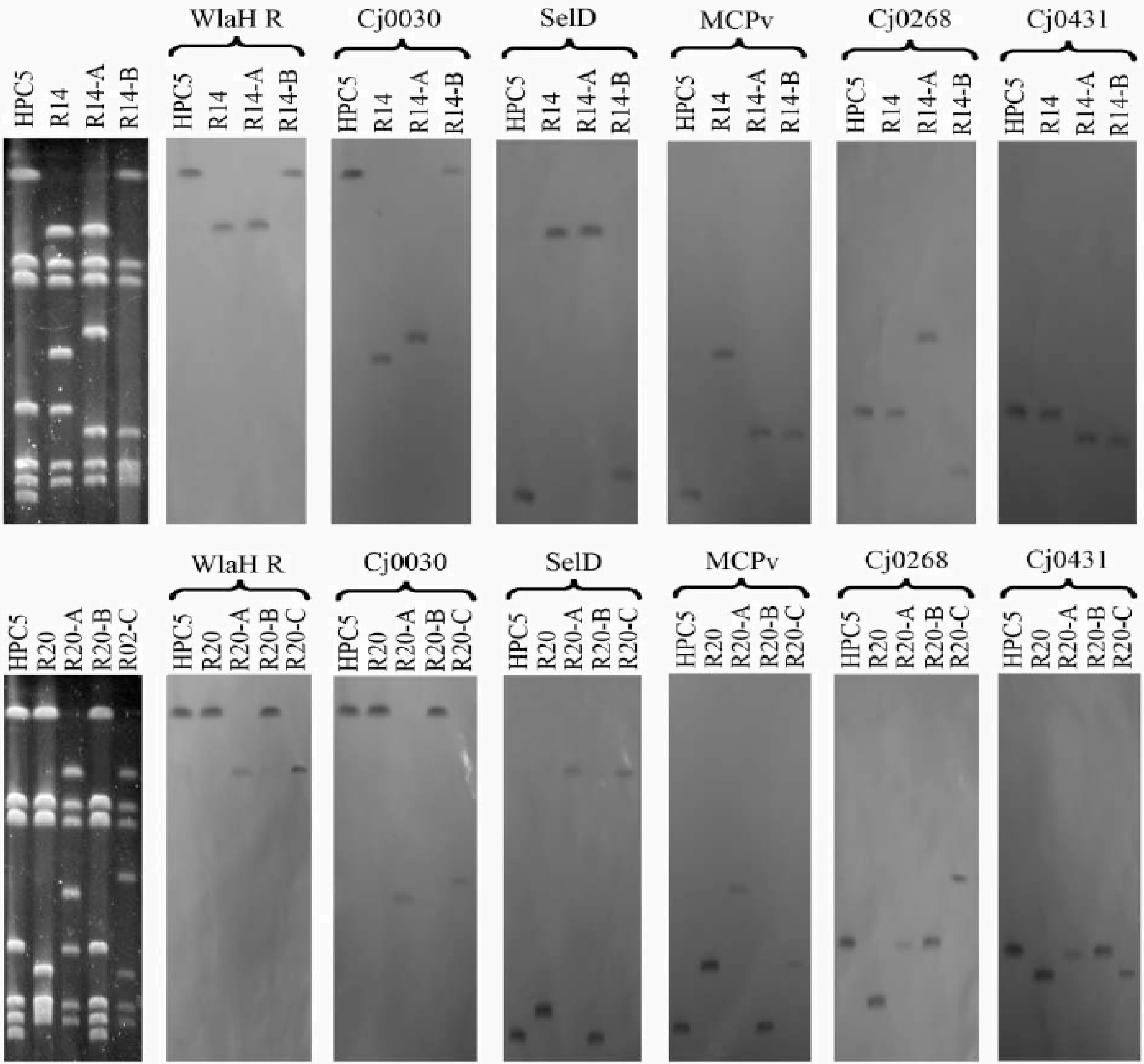

HPC5 R14 R14-A R14-B
WlaH R
Cj0030
SelD
MCPv
Cj0268
Cj0431
HPC5 R20 R20-A R20-B R02-C
HPC5 R20 R20-A R20-B R20-C
