# Supplementary material for: Genome Dynamics of Campylobacter jejuni in Response to Bacteriophage Predation
Source: PLoS Pathog. 2007 Aug 24;3(8):e119. doi: 10.1371/journal.ppat.0030119 (PMC1950947; doi:10.1371/journal.ppat.0030119)
Supplement: Supplementary file 3 [file ppat.0030119.sg003.pdf]

FigureS3

A

```
         5         15         25         35         45         55
AAGCTTATTA TCTTAATCTT TTTAACTCTT ACTTTCTTAT ATACAGCCTT ACGTCCTAAC

        65         75         85         95        105        115
TTAGGCAAAC ACGAAAGCGA ACCTAAAATA AAAAATATCA AAATTTTTCA TTTAATTTGT

       125        135        145        155        165        175
GGCTTAACTT TAGGATTTTA TGATGGATTT TTAGGGCCTG GAACGGGTTC ATTTTGGATT

       185        195        205        215        225        235
TTTGCTTGTG TGATGCTACT AGGTTTTAAT ATGAGAAAAG CAAGCATTAA TACTAAAATT

       245        255        265        275        285        295
TTAAATTTCA CTAGTAATAT CATTGCTCTT GCTATATTTT TATGGCAATA CGAACTTTTA

       305        315        325        335        345        355
TGGGCTGTAG GCTTGCTTAT GGGCGTAGGA CAAGTTTTAG GAGCTTATTT AGGCTCAAAA

       365        375        385        395        405        415
CTTGTATTAA AAACTAATGG AAAGTTTATA AAAACTTTAT TTTTAATCGT TGTAGGAGCT

       425        435        445        455        465        475
ACTATCATTA AAGTAGCTTG GGATTATTTT AGTTAAAAAT TGACCCCCGA ACTAAGCTCC

       485        495        505        515        525        535
CACATAGGTA AAAATATACC TAAAGCCAAA AACAAAACTA AAAACGCTAC AAATAAAGTC

       545        555        565        575        585        595
ATCATAGGCT CTAAAAAAAC CAAAAATCTA TCCATTAAAT TTTCACTTTT TTGTTGATAA

       605        615        625        635        645        655
TATTGGCAAT TCTTCACTAA CACTCAATGA CTTTTTACTT TCCGATCTAA GCGTAAATAT

       665        675        685        695        705        715
CCCAATTATC CCAAAATGTC CGTTTTGTCC CAAAAAAGTG ATCAAATGCT GTTTAAAAGG

       725        735        745        755        765        775
CGGATAATAC GAGTTTTAAT TTTAATTTTT ATATCCCAAA AGTGTTTTTA CTCTCTAGGA

       785        795        805        815        825        835
TAGTTACATT TTTACTTAAA GTATCCTTAT GTTTTCTTAA TCATGTAACT ATCCC
```

B

Cj1468 [Campylobacter jejuni 11168] Expect = 0.0

Cj1470c (C-Terminus) [Campylobacter jejuni 11168] Expect = 3e-65

CJE0213 [Campylobacter jejuni RM1221] Expect = 3e-52
