# Supplementary material for: Genome Dynamics of Campylobacter jejuni in Response to Bacteriophage Predation
Source: PLoS Pathog. 2007 Aug 24;3(8):e119. doi: 10.1371/journal.ppat.0030119 (PMC1950947; doi:10.1371/journal.ppat.0030119)
Supplement: Supplementary file 4 [file ppat.0030119.sg004.pdf]

Figure S4

A

```
....|....| ....|....| ....|....| ....|....| ....|....| ....|....|
    5         15         25         35         45         55
TTTATCAAAA TAAAGATATT GCAGAAAGCA GAAGAAAACT TGCATTAGAA ATAGAACAAA

....|....| ....|....| ....|....| ....|....| ....|....| ....|....|
    65        75         85         95        105        115
AAATACAGAA AGGATATCCT AAAGCCTATG GCTTAATTAT AGATATAATT CAGCTTTTGG

....|....| ....|....| ....|....| ....|....| ....|....| ....|....|
   125        135        145        155        165        175
AAGATAATTA TGATGTAAAT CTTTTTGAAA ATCAATGCAT TAAGTATTAT GAAGAAGCTC

....|....| ....|....| ....|....| ....|....| ....|....| ....|....|
   185        195        205        215        225        235
AAAAGTTAAA AACCATTAAA TCCAAGCAAA AAAGAATTGA TTATATAAAT GATTTAATAA

....|....| ....|....| ....|....| ....|....| ....|....| ....|....|
   245        255        265        275        285        295
AAGAAGCTGA AATAAATCCT AAAATGGATG AGAAATTTGT TAATTTTTGG AAAAATAAAG

....|....| ....|....| ....|....| ....|....| ....|....| ....|....|
   305        315        325        335        345        355
TAAAAGAAAT ACAATAATTA CATTTTAAGT CTAAGGCTTG TAGAAAAATA AAATGCAACC

....|....| ....|....| ....|....| ....|....| ....|....| ....|....|
   365        375        385        395        405        415
AAAAAGTATG ATTTTATTTT TCTATGGCTA CCTTTTATTT TTCCGCCGAC ATCTACGATA

....|....| ....|....| ....|....| ....|....| ....|....| ....|....|
   425        435        445        455        465        475
ATAAAAACCA ATCCCCCTAA AAGTTCACCT TTATTTTTAA GATAAATACC AAATTTACTC

....|....| ....|..
   485        495
CCTATCCTTA GAGCAAT
```

B

ORF 0656 [Campylobacter jejuni CF93-6] Expect = 7e-52

Cj0167c (N-terminus) [Campylobacter jejuni 11168] Expect = 1e-6
