# Supplementary material for: Genome Dynamics of Campylobacter jejuni in Response to Bacteriophage Predation
Source: PLoS Pathog. 2007 Aug 24;3(8):e119. doi: 10.1371/journal.ppat.0030119 (PMC1950947; doi:10.1371/journal.ppat.0030119)
Supplement: Supplementary file 5 [file ppat.0030119.sg005.pdf]

Figure S5

A

```
....|....| ....|....| ....|....| ....|....| ....|....| ....|....|
       5         15         25         35         45         55
AAGCTTTAGC TACTTTAAGA ATGAAATTTT CAATCGAAAA ATAAATGTTC TATTAGAATT

....|....| ....|....| ....|....| ....|....| ....|....| ....|....|
      65         75         85         95        105        115
TTTACTCCTA GTGTAAGGGG TATTTTAAAA AGAACATGTA TCCAAATAAT AGGATTTTAA

....|....| ....|....| ....|....| ....|....| ....|....| ....|....|
     125        135        145        155        165        175
AGCATATAAA AATGACTTTT TATCTTTCCC GATCTAAAGC GTAAAATATC CCAATTATCC

....|....| ....|....| ....|....| ....|....| ....|....| ....|....|
     185        195        205        215        225        235
CAAAATGTCC GTTTTGTCCC AAAAAAGTGA TCAAATGCTG TTTAAAAGGC GGATAAATAC

....|....| ....|....| ....|....| ....|....| ....|....| ....|....|
     245        255        265        275        285        295
GAGTTTTAAT TTTAATTTTT ATATCCCAAA AGTGTTTTTA CTCTCTAGGA TAGTTACATT

....|....| ....|....| ....|....| ....|....| .
     305        315        325        335
TTTACTTAAA GTATCCTTAT GTTTTCTTAA TCATGTAACT A
```

B

miaA [Campylobacter jejuni 11168] Expect = 2e-17

Cj0167c (C-Terminus) [Campylobacter jejuni 11168] Expect = 9e-17

CJE0213 [Campylobacter jejuni RM1221] Expect = 4e-62
