# Supplementary material for: Genome Dynamics of Campylobacter jejuni in Response to Bacteriophage Predation
Source: PLoS Pathog. 2007 Aug 24;3(8):e119. doi: 10.1371/journal.ppat.0030119 (PMC1950947; doi:10.1371/journal.ppat.0030119)
Supplement: Supplementary file 6 [file ppat.0030119.sg006.pdf]

Figure S6

A

```
....|....| ....|....| ....|....| ....|....| ....|....| ....|....|
    5         15         25         35         45         55
TTATCAAAAT AAAGATATTG CAGAAAGCAG AAGAAAACTT GCATTAGAAA TAGAACAAAA

....|....| ....|....| ....|....| ....|....| ....|....| ....|....|
   65         75         85         95        105        115
AATACAGAAA GGATATCCTA AAGCCTATGG CTTAATTATA GATATAATTC AGCTTTTGGA

....|....| ....|....| ....|....| ....|....| ....|....| ....|....|
   125        135        145        155        165        175
AGATAATTAT GATGTAAATC TTTTTGAAAA TCAATGCATT AAGTATTATG AAGAAGCTCA

....|....| ....|....| ....|....| ....|....| ....|....| ....|....|
   185        195        205        215        225        235
AAAGTTAAAA ACCATTAAAT CCAAGCAAAA AAGAATTGAT TATATAAATG ATTTAATAAA

....|....| ....|....| ....|....| ....|....| ....|....| ....|....|
   245        255        265        275        285        295
AGAAGCTGAA ATAAATCCTA AAATCGATGA GAAATTTGTT AATTTTTGGA AAAATAAAGT

....|....| ....|....| ....|....| ....|....| ....|....| ....|....|
   305        315        325        335        345        355
AAAAGAAATA CAATAATTAC ATTTTAAGTT TAAGGTTTGT AGAAAAATAA AATGCAACCA

....|....| ....|....| ....|....| ....|....| ....|....| ....|....|
   365        375        385        395        405        415
AAAAGTATCA TTTTATTTTT CTATGGTTAC ATTTCATTTT TCCGCCGATA TCTTCCACTT

....|....| ....|....| ....|....| ....|....| ....|....| ....|....|
   425        435        445        455        465        475
TTCATCGCCA CACTGAGCAT AGAAATTACC AAAGAATCAA AAATGTCGAT CATTTTGAAA

....|....| ....|....| ....|....| ....|....| ....|....| ....|....|
   485        495        505        515        525        535
GCCTTTCTTA GCTCAAAAAC AGAACCAATT AAACTAATAA GTCTTTTATC TGATAAATTA

....|....| ....|....| ....|.
   545        555        565
CAATTTCTTT CTCCCGGCTC GCAATA
```

B

ORF 0656 [Campylobacter jejuni CF93-6] Expect = 7e-52

Cj1470c (N-terminus) [Campylobacter jejuni 11168] Expect = 4e-06
