# Supplementary material for: Genome Dynamics of Campylobacter jejuni in Response to Bacteriophage Predation
Source: PLoS Pathog. 2007 Aug 24;3(8):e119. doi: 10.1371/journal.ppat.0030119 (PMC1950947; doi:10.1371/journal.ppat.0030119)
Supplement: Supplementary file 7 [file ppat.0030119.sg007.pdf]

Figure S7

A

```
....|....| ....|....| ....|....| ....|....| ....|....| ....|....|
    5          15         25         35         45         55
TGTCTATAAG CTCATCACTT GGCACATTTT GAGGCAATTC ACATTTGCCG CATTTACATT

....|....| ....|....| ....|....| ....|....| ....|....| ....|....|
    65         75         85         95        105        115
TAAATTCGCT TTCTTTAAAA TAAGGGTTAT TTTTCATTTT CTTCTTCTCC TTCTGGTTTT

....|....| ....|....| ....|....| ....|....| ....|....| ....|....|
   125        135        145        155        165        175
GAAAAAATTT CTATATTAAA TTTAAGCCTA TAAGCAAATA AAGAACTGCT TATAAAACCA

....|....| ....|....| ....|....| ....|....| ....|....| ....|....|
   185        195        205        215        225        235
CTTTCGATAC TGCTTAAAAG CTTTTAGCTT GTATCAAATT CAAAAAGTTT TAGTCTTAAT

....|....| ....|....| ....|....| ....|....| ....|....| ....|....|
   245        255        265        275        285        295
TCATCTATTT TTTTAATCAA AGAATCTTTA TCTTTATTAA AAGTATGAGA ACTAAAAATC

....|....| ....|....| ....|....| ....|....| ....|....| ....|....|
   305        315        325        335        345        355
AATGAAAAAT TCACACTATC TTTATGATGA TTAATACTTG TTAGGCTTTC AAGCATTAGA

....|....| ....|....| ....|....| ....|....| ....|....| ....|....|
   365        375        385        395        405        415
TAAAGTCCGC CTTGTTTGAC ATCTTCGTAA TTTGCTATGC CTATCGCATT AAAATGCTTT

....|....| ....|....| ....|....| ....|....| ....|....| ....|....|
   425        435        445        455        465        475
AATAAATCTT TGCTAACACT ATCTAAAGTC AAAACCTGTC CTTTCTTTGC ATAATTTTAA

....|....| ....|....| ....|....| ....|....| ....|....| ....|....|
   485        495        505        515        525        535
GTCTTAAAGA GCTTAAATTT CCTTCTTTAT CAATCAAAGG TGCATTTTCA ATTCGTTTTA

....|....| ....|....| ....|....| ....|....| ....|....| ....|....|
   545        555        565        575        585        595
AAGCATTATC TAGAAGGATT TTATCTTCAT CATCTAAGAC GATTTTAAGA TAAATTTTTA

....|....| ....|....| ....|....| ....|....| ....|....| ....|....|
   605        615        625        635        645        655
TGCGAATGTA TGCAAAATCC TCCCAAATAA AATCAGCAAT ATCTTTATTT TTGCAAATAT

....|....| ....|....| ....|....| ....|....| ....|....| ....|....|
   665        675        685        695        705        715
CATCTGCATC TTTTGCTGCT TTAGCTACTA CTTCTCTTGT AATTTCATCG ATATTTGCTA

....|....| ....|....| ....|....| ....|....| ....|....| ....|....|
   725        735        745        755        765        775
AGTTTTGTTG TGCTCTTTTC ATCAGTGCTA ACTCTTTTAA AGTAAGAGCT AGCATTTTAG
```

B

CJE0241 [Campylobacter jejuni RM1221] Expect = 5e-15

Unknown Sequence
