# Supplementary material for: Genome Dynamics of Campylobacter jejuni in Response to Bacteriophage Predation
Source: PLoS Pathog. 2007 Aug 24;3(8):e119. doi: 10.1371/journal.ppat.0030119 (PMC1950947; doi:10.1371/journal.ppat.0030119)
Supplement: Supplementary file 8 [file ppat.0030119.sg008.pdf]

Figure S8

A

```
....|....| ....|....| ....|....| ....|....| ....|....| ....|....|
    5         15         25         35         45         55
TGAAATCCCT TGTTGCAAGG TGGAATTTAC TTCCTCCGCC AATTTAAAAG AAGGAGAATA

....|....| ....|....| ....|....| ....|....| ....|....| ....|....|
   65         75         85         95        105        115
ACAGGTTTAA TAAAGTACCT CAAGTAATTG AACAAGCCAA TTGTTTTATA GATGGGTATG

....|....| ....|....| ....|....| ....|....| ....|....| ....|....|
  125        135        145        155        165        175
GTTATGCTGG GGTAGCAAGA GATATCACTT TGCCTATTAT AGAACAAGAA GTATTAGAAA

....|....| ....|....| ....|....| ....|....| ....|....| ....|....|
  185        195        205        215        225        235
GCAAAGGAGC GCTGAGTGCA AATTATGGCT CGGGTGGATT TAAAGCAATG CAATGCTCTT

....|....| ....|....| ....|....| ....|....| ....|....| ....|....|
  245        255        265        275        285        295
TTAACATTAG CGACATGGGA GAACAAGCTT TTGAAGCTTT TGGAGTAAAT ACTTTTTCTA

....|....| ....|....| ....|....| ....|....| ....|....| ....|....|
  305        315        325        335        345        355
AAACAAAAAT CCCACTTGTT TTTAAAGCAA GTATTCATCA AAGCGGATCA GGAAAACAAG

....|....| ....|....| ....|....| ....|....| ....|....| ....|....|
  365        375        385        395        405        415
TGCCTTTTGT AGTGGAGTTA AATGGAGAAT TTACATCTAT GACTCCCCCT TCTTTTGCAA

....|....| ....|....| ....|....| ....|....| ....|....| ....|....|
  425        435        445        455        465        475
CAGGTGGCGA ATTTACAAGT GAAACAAAAA CCCATGCGCC TTTTATAAAA ATCACCCTGG

....|....| ....|....| ....|....| ....|....| ....|....| ....|....|
  485        495        505        515        525        535
ATGGCAAAAG ACTATTTTTA GCTGATATAA AATACCCTAT TCTAGACTTT AATGGCATTG

....|....| ....|....| ....|....| ....|....|
  545        555        565        575
ATCAAATGGC CAACGTAAAA GCCAATTTCA GCCTATAAGG
```

B

Phage major tail tube protein [Campylobacter jejuni 260.94] Expect = 8e-25
