# Supplementary material for: Genome Dynamics of Campylobacter jejuni in Response to Bacteriophage Predation
Source: PLoS Pathog. 2007 Aug 24;3(8):e119. doi: 10.1371/journal.ppat.0030119 (PMC1950947; doi:10.1371/journal.ppat.0030119)
Supplement: Supplementary file 9 [file ppat.0030119.sg009.pdf]

Figure S9

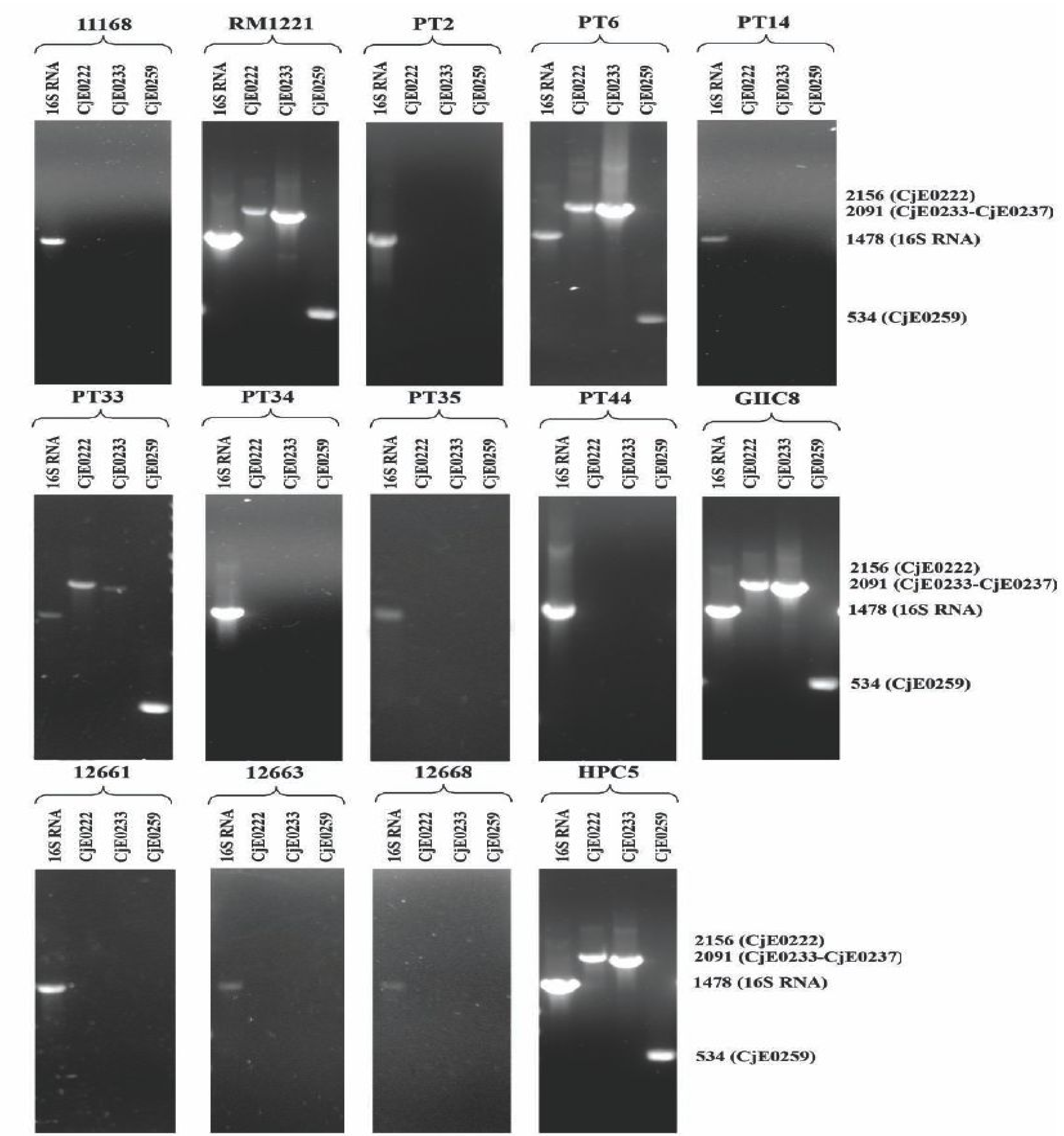
11168
RM1221
PT2
PT6
PT14
PT33
PT34
PT35
PT44
GIIC8
12661
12663
12668
HPC5
16S RNA
CjE0222
CjE0233
CjE0259
2156 (CjE0222)
2091 (CjE0233-CjE0237)
1478 (16S RNA)
534 (CjE0259)
