# Supplementary material for: Genome Dynamics of Campylobacter jejuni in Response to Bacteriophage Predation
Source: PLoS Pathog. 2007 Aug 24;3(8):e119. doi: 10.1371/journal.ppat.0030119 (PMC1950947; doi:10.1371/journal.ppat.0030119)
Supplement: Supplementary file 10 [file ppat.0030119.sg010.pdf]

Figure S10

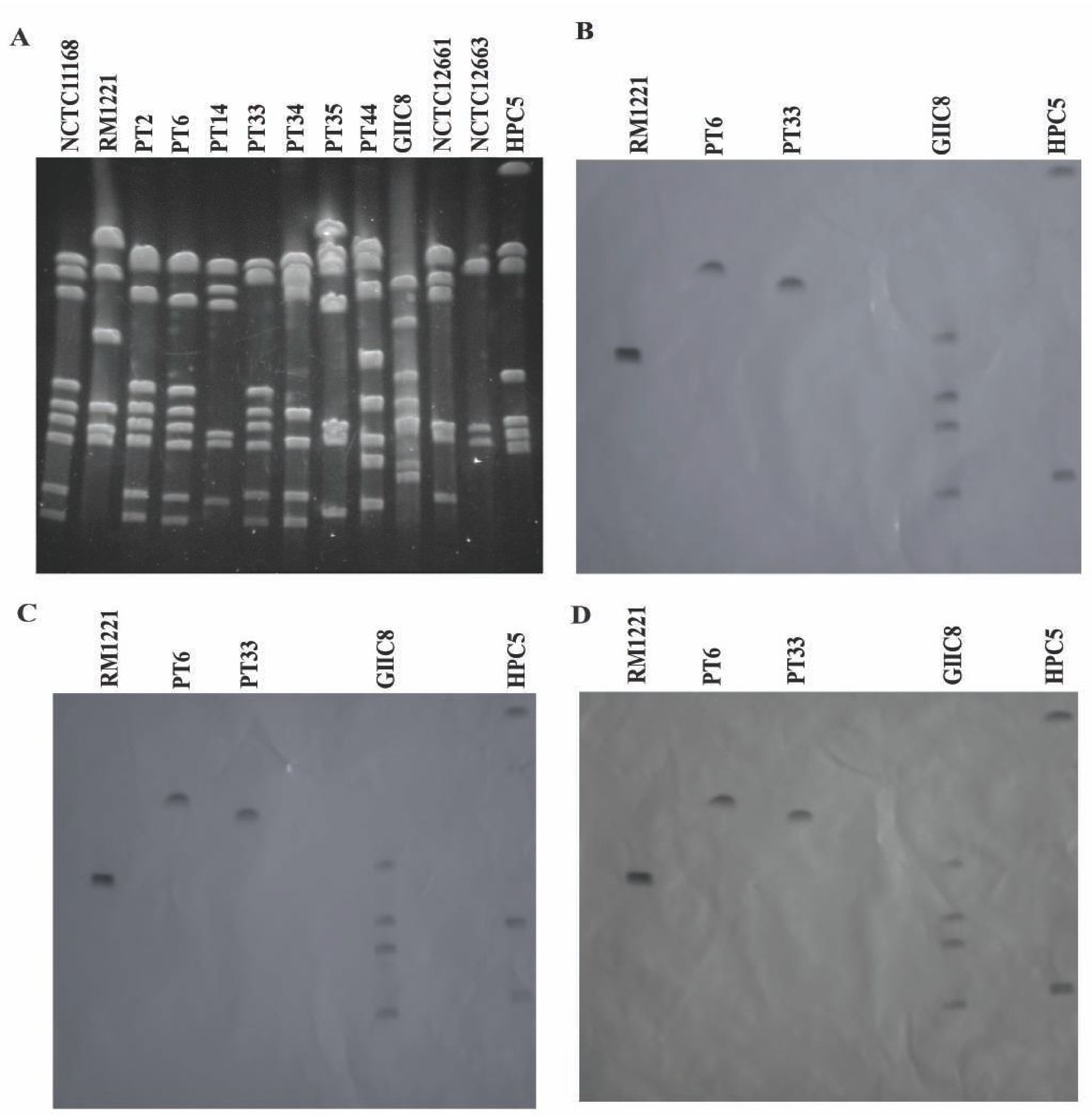
A
NCTC11168
RM1221
PT2
PT6
PT14
PT33
PT34
PT35
PT44
GIIC8
NCTC12661
NCTC12663
HPC5
B
RM1221
PT6
PT33
GIIC8
HPC5
C
RM1221
PT6
PT33
GIIC8
HPC5
D
RM1221
PT6
PT33
GIIC8
HPC5
