# Supplementary material for: Genome Dynamics of Campylobacter jejuni in Response to Bacteriophage Predation
Source: PLoS Pathog. 2007 Aug 24;3(8):e119. doi: 10.1371/journal.ppat.0030119 (PMC1950947; doi:10.1371/journal.ppat.0030119)
Supplement: Supplementary file 11 [file ppat.0030119.st001.pdf]

| Primer | Sequence (5' → 3') | Location / Use |
|---|---|---|
| CjE0215 F | AAA GGA TAT ACT TGC GAG TG | Mu-Like Prophage Gene |
| CjE0215 R | AGT TCT CCA ATT ACT ACA CC | Mu-Like Prophage Gene |
| CjE0222 F | TCT TGC TTT CTT AAA GCG TC | Mu-Like Prophage Gene |
| CjE0222 R | GGT GTT ATT TTA GGA CTA GC | Mu-Like Prophage Gene |
| CjE0225 F | AAA TCT TTA AGA GCG TCC TG | Mu-Like Prophage Gene |
| CjE0225 R | CAA ACT TGA AAA TGG CGA AG | Mu-Like Prophage Gene |
| CjE0231 F | CCT CAA TAT TTG GCT TAT CC | Mu-Like Prophage Gene |
| CjE0231 R | TTT ATT GCT GCA AGA GCA AG | Mu-Like Prophage Gene |
| CjE0233 F | GCT AGG TTC TAT GTT TGT AG | Mu-Like Prophage Gene |
| CjE0237 R | CTC TTT AAC CAC ATC AAC AC | Mu-Like Prophage Gene |
| CjE0241 F | TAT GGA TAA GTC CAT CTT GC | Mu-Like Prophage Gene |
| CjE0241 R | TGA ATT GCC TCA AAA TGT GC | Mu-Like Prophage Gene |
| CjE0244 F | GGA AAA TGA TGC CTT AAT GG | Mu-Like Prophage Gene |
| CjE0244 R | TAA ATC CTT GCA AGC ATA GG | Mu-Like Prophage Gene |
| CjE0250 F | TGC TTA AAA TCA GCG AGA AC | Mu-Like Prophage Gene |
| CjE0250 R | AAA CTC GCT TTG CTC TAA AC | Mu-Like Prophage Gene |
| CjE0259 F | AGT TTG GCT AGG AAT TTG AG | Mu-Like Prophage Gene |
| CjE0259 R | GGT ATT AGT GAA GTT CAA GC | Mu-Like Prophage Gene |
| CjE0270 F | AAA GCT TCC ATG TCA AAC TC | Mu-Like Prophage Gene |
| CjE0270 R | GCT TTT AAT GTA AGC ACA GG | Mu-Like Prophage Gene |
| CjE0270 F2 | CCT TTA ATC ATA TCA GGC TTA CC | CjE0270-End Sequencing |
| CjE0270 F3 | CGG TAG TAA TAA TTT CAC TC | CjE0270-End Sequencing |
| CjE0270 F4 | GCT GGA TAT GGT GCA ATA AAT AGC | CjE0270-End Sequencing |
| CjE0213i F | GCA AAA ATC CCA AAT AAT AGG | CjE0213-End Inverse PCR |
| CjE0213i R | TTT AGG GAT AGT TAC ATG TTC | CjE0213-End Inverse PCR |
| CjE0227i F | ATT CAT AGA ACT TGC CAC AC | Partial Copy Inverse PCR |
| CjE0227i R | CAG CTA TTG TGG ATC TTT AC | Partial Copy Inverse PCR |
| CjE0241i F | GCT ATT TTT GAA GGA CTT AAA AAC C | Partial Copy Inverse PCR |
| CjE0241i R | GTT CTA ATT GCT CTT TGT GG | Partial Copy Inverse PCR |
| CjE0270i F | CAA ATT GCA GAA GAA AAG CG | CjE0270-End Inverse PCR |
| CjE0270i R | GCT TCT GCA TAG TCA TCT GG | CjE0270-End Inverse PCR |
| Cj1468 F | AGA TTC TAT TGT AGG TGG AG | CjE0213 Adjacent Sequence |
| Cj1468 R | TAA TAG TAG CTC CTA CAA CG | CjE0213 Adjacent Sequence |
| Cj0166 F | AGT GCT ATT TTT CCA AGT GC | CjE0213 Adjacent Sequence |
| Cj0166 R | TCC TCT ATG ATT GTA GCA TC | CjE0213 Adjacent Sequence |
| 227 Unk F | AGG CTG AAA TTG GCT TTT ACG | CjE0227 Adjacent Sequence |
| 227 Unk R | CTT TCT CAC AGA GAG TGC AAA G | CjE0227 Adjacent Sequence |
| 241 Unk F | CTT CTT CTC CTT CTG GTT TTG | CjE0241 Adjacent Sequence |
| 241 Unk R | CCT AAA ATG CTA GCT CTT AG | CjE0241 Adjacent Sequence |
| Cj0167 F | GAG CAA TGA TAC ATA AAA TGA AGG | CjE0270 Adjacent Sequence |
| Cj0167 R | ATG CTT TTG CTG TAT CAC TAT GC | CjE0270 Adjacent Sequence |
| CtsF F | CAT CGC AAC ACT GAG CAT AG | CjE0270 Adjacent Sequence |
| CtsF R | AGA GCT AGC TTT GTT GTG TG | CjE0270 Adjacent Sequence |
| Cj0752 F | AGC AAG GAT TAA TCT TCT CG | Gene Adjacent to SmaI Site |
| Cj0752 R | AGC TAA TTC GAA AGG CTT TG | Gene Adjacent to SmaI Site |
| WlaH F | TTT CTC TGT TGT AAC ATG GC | Gene Adjacent to SmaI Site |
| WlaH R | AGC TTT AGT GCT TTT AGT GC | Gene Adjacent to SmaI Site |
| Cj0030 F | AAA AGC GTA GAA ACA GAA GC | Gene Adjacent to SmaI Site |
| Cj0030 R | CCT TTA TAT GCT TCC TGT ATA GG | Gene Adjacent to SmaI Site |
| AnsA F | TTG CTA TTT TAG GAA CAG GC | Gene Adjacent to SmaI Site |
| AnsA R | TTC ATC TGA ATC ACT CAC AG | Gene Adjacent to SmaI Site |
| RibD F | ACA AGG TAG GCA AAT GAA AG | Gene Adjacent to SmaI Site |
| RibD R | AAT GCC ATA AGT ATC ACT CC | Gene Adjacent to SmaI Site |
| Cj1621 F | ACT TTT TGT TGC AGG AGT AG | Gene Adjacent to SmaI Site |
| Cj1621 R | TAG GTG CGA TAT TGG TAT TG | Gene Adjacent to SmaI Site |
| Cj1505 F | TTG CTT TAC CAA AAA GCT CG | Gene Adjacent to SmaI Site |
| Cj1505 R | GAT TGT CGC AAT CTA TCT TG | Gene Adjacent to SmaI Site |
| SelD F | AAA AGT CCT CCT GAA GTT TG | Gene Adjacent to SmaI Site |
| SelD R | AAC TCC TGC TTT ACT TAG TG | Gene Adjacent to SmaI Site |
| MCPv F | TAC ACA ACA GCC AAA ATA GC | Gene Adjacent to SmaI Site |
| MCPv R | AAC GAT GAG GGT ATT ATT GC | Gene Adjacent to SmaI Site |
| Cj0268 F | TTT AGG CTG TTT GCG ATT TC | Gene Adjacent to SmaI Site |
| Cj0268 R | GGT TTT GGA AAA TTC TCT CC | Gene Adjacent to SmaI Site |
| Cj0431 F | AGA CAA TCT ACT AGA AGG TG | Gene Adjacent to SmaI Site |
| Cj0431 R | GCT ATA TTT TAG AGC ACT CG | Gene Adjacent to SmaI Site |
| MurD F | TTA AAG ACT TTT CCA CGC TC | Gene Adjacent to SmaI Site |
| MurD R | TTT GGA TAC GGA AAA ACC AC | Gene Adjacent to SmaI Site |
| Cj0742 F | ATT TTC ACA TGC TGA AGA GC | Gene Adjacent to SmaI Site |
| Cj0742 R | ATA CCC ATT GCA TCC ATA AG | Gene Adjacent to SmaI Site |
| 16S F | TTA TGG AGA GTT TGA TCC TG | 16S rRNA Gene |
| 16S R | TTA CCT TGT TAC GAC TTC AC | 16S rRNA Gene |

Supporting Table 1

Table indicating the PCR primers used during this study and their primary functions. All primers are designed and named based on the *C. jejuni* NCTC11168 or RM1221 genes they are located within. Primers used for MLST analysis (not shown here) were according to Dingle *et al.* (2001).
